# Trends on *Aspergillus* Epidemiology—Perspectives from a National Reference Laboratory Surveillance Program

**DOI:** 10.3390/jof7010028

**Published:** 2021-01-06

**Authors:** Raquel Sabino, Paulo Gonçalves, Aryse Martins Melo, Daniela Simões, Mariana Oliveira, Mariana Francisco, Carla Viegas, Dinah Carvalho, Carlos Martins, Teresa Ferreira, Cristina Toscano, Helena Simões, Cristina Veríssimo

**Affiliations:** 1 Infectious Diseases Department, National Health Institute Dr. Ricardo Jorge, 1649-016 Lisbon, Portugal; paulo.goncalves@insa.min-saude.pt (P.G.); arysemartins@gmail.com (A.M.M.); dsofia.simoes@gmail.com (D.S.); mariana.luisa.05@gmail.com (M.O.); marianinha.francisco@gmail.com (M.F.); helena.simoes@insa.min-saude.pt (H.S.); cristina.verissimo@insa.min-saude.pt (C.V.); 2 European Programme for Public Health Microbiology Training (EUPHEM), European Centre for Disease Prevention and Control, 16973 Solna, Sweden; 3 Programa de Pós-Graduação em Microbiologia e Parasitologia, Instituto de Biologia, Universidade Federal de Pelotas, Avenida Eliseu Maciel, Pelotas 96010-610, Brazil; 4 H&TRC—Health & Technology Research Center, ESTeSL—Escola Superior de Tecnologia da Saúde, Instituto Politécnico de Lisboa, 1990-096 Lisbon, Portugal; carla.viegas@estesl.ipl.pt; 5 NOVA National School of Public Health, Public Health Research Centre, Universidade NOVA de Lisboa, 1600-560 Lisbon, Portugal; 6 Comprehensive Health Research Center (CHRC), 1169-056 Lisbon, Portugal; 7 Centro Hospitalar Universitário Lisboa Norte EPE, 1649-028 Lisbon, Portugal; dinah.carvalho@chln.min-saude.pt (D.C.); cmartins092@gmail.com (C.M.); 8 Centro Hospitalar Universitário Lisboa Central, 1050-099 Lisbon, Portugal; teresa.ferreira@gmail.com; 9 Microbiology Laboratory, Centro Hospitalar Lisboa Ocidental, Hospital Egas Moniz, 1349-019 Lisbon, Portugal; ctoscano@chlo.min-saude.pt

**Keywords:** *Aspergillus*, surveillance, molecular epidemiology, cryptic species, Azole resistance mutations

## Abstract

Identification of *Aspergillus* to species level is important since sibling species may display variable susceptibilities to multiple antifungal drugs and also because correct identification contributes to improve the knowledge of epidemiological studies. Two retrospective laboratory studies were conducted on *Aspergillus* surveillance at the Portuguese National Mycology Reference Laboratory. The first, covering the period 2017–2018, aimed to study the molecular epidemiology of 256 *Aspergillus* isolates obtained from patients with respiratory, subcutaneous, or systemic infections and from environmental samples. The second, using our entire collection of clinical and environmental *A. fumigatus* isolates (*N* = 337), collected between 2012 and 2019, aimed to determine the frequency of azole-resistant *A. fumigatus* isolates. *Aspergillus fumigatus* sensu stricto was the most frequent species in both clinical and environmental samples. Overall, and considering all *Aspergillus* sections identified, a high frequency of cryptic species was detected, based on beta-tubulin or calmodulin sequencing (37% in clinical and 51% in environmental isolates). Regarding all *Fumigati* isolates recovered from 2012–2019, the frequency of cryptic species was 5.3% (18/337), with the identification of *A. felis* (complex), *A. lentulus*, *A. udagawae*, *A. hiratsukae*, and *A. oerlinghauensis*. To determine the frequency of azole resistance of *A. fumigatus*, isolates were screened for azole resistance using azole-agars, and 53 possible resistant isolates were tested by the CLSI microdilution reference method. Nine *A. fumigatus* sensu stricto and six *Fumigati* cryptic isolates showed high minimal inhibitory concentrations to itraconazole, voriconazole, and/or posaconazole. Real-time PCR to detect *cyp51A* mutations and sequencing of *cyp*51A gene and its promoter were performed. The overall frequency of resistance to azoles in *A. fumigatus* sensu stricto was 3.0%. With this retrospective analysis, we were able to detect one azole-resistant G54R mutant *A. fumigatus* environmental isolate, collected in 2015. The TR_34_/L98H mutation, linked to environmental transmission route of azole resistance, was the most frequently detected mutation (*N* = 4; 1.4%). Our findings underline the demand for correct identification and susceptibility testing of *Aspergillus* isolates.

## 1. Introduction

Invasive aspergillosis (IA) affects about 300,000 patients per year and more than 30 million patients are at risk [1]. The genus *Aspergillus* is composed of several hundred species, some of which are considered to be potentially pathogenic, causing serious infections with a fatality rate that can reach 50% if treated and more than 99% if not treated [1]. The main pathologies associated with infections by *Aspergillus* affect the lungs, including allergic bronchopulmonary aspergillosis (ABPA), and chronic pulmonary aspergillosis (CPA), but the infection can also spread to other organs leading to the development of IA [2]. Patients at high risk of acquiring IA generally suffer from severe granulocytopenia (leukemia, bone marrow, or solid organ transplant patients). In addition, prolonged use of corticosteroids, diabetes, severe burns, and major surgery are considered predisposing risk factors for IA [3]. 

Molecular studies [4,5] have shown the limitation of conventional identification through morphological characteristics to distinguish *Aspergillus* species. In fact, an isolate identified using morphological methodologies can only be included in one of the “species sections”, such as *Fumigati*, *Flavi*, *Nidulantes*, *Usti,* or *Terrei*. Species that are morphologically identical and only distinguishable from each other using molecular methodologies are called cryptic species. Molecular differences also reflect a difference in susceptibility to antifungals, with cryptic species being in general less susceptible [4].

The *Aspergillus* species most frequently isolated in the clinical context are *A. fumigatus*, *A. flavus*, *A. niger*, and *A. terreus*. The *Fumigati* section, composed of *A. fumigatus* sensu stricto and its cryptic species, is the most frequently isolated from clinical samples and is also frequently isolated from environmental sources. Moreover, the prevalence of clinical isolates of the *Fumigati* section with azole resistance has been increasing [6]. The proposed reasons for this include the development of resistance by prolonged antifungal prophylaxis or therapy. However, the inhalation of environmental strains resistant to antifungals is currently one of the biggest and most recent concerns of the scientific community [6,7,8]. 

It has been shown that azole-resistant *A. fumigatus* isolates have, for the most part, a resistance mechanism mediated by the *cyp51A* gene. Depending on the specific mutation, they may show resistance to one azole or to any azole [6]. TR_34_/L98H and TR_46_/Y121F/T289A are the most common mutations associated with environmental pan-azole resistance. However, other mutations in the promoter of the *cyp51A* gene, as TR_53_, TR_46_^3^, and TR_46_^4^, have also been described as from environmental origin [9]. A recently described mutation in the promoter of the *cyp51A* gene is TR_120,_ probably more associated with prolonged azole exposure [10]. Other mutations at various positions of the *cyp51A* gene such as G54, M220, P216, G138, and G448 have also been associated with resistance, of which the G54 and M220 mutations are the most common [11,12].

Azoles are the first line of prophylaxis and treatment of infections by *A. fumigatus* and, therefore, there is a high concern inherent to treatment failure. Resistance to triazoles can severely limit treatment options and be associated with worse patient prognosis [13].

The European Center for Disease Prevention and Control (ECDC) published in 2013 [14] a recommendation for epidemiological surveillance to be carried out, to collect information at the local level, both in clinical and environmental context. In Portugal, few reports on *Aspergillus* prevalence and resistance have been published [15,16]. As such, we hypothesized that the reported prevalence of azole-resistant *A. fumigatus* may not represent the true prevalence of azole resistance and, therefore, surveillance studies are warranted. 

Here, we report the results from the laboratory-based national surveillance program for *Aspergillus* that is established in our country since 2012, coordinated by the National Reference Laboratory for Mycology at the National Institute of Health Dr. Ricardo Jorge in Lisbon. Our laboratory provides reference diagnostic services to several hospitals from the National Health Service and for with a wide range of microbiological and clinical specialties from different regions of Portugal. In the context of this program, our laboratory receives clinical and environmental specimens or isolates for identification and antifungal susceptibility profiling of *Aspergillus*. Thus, with the two retrospective laboratory studies here presented, we are able to evaluate the frequency and diversity of *Aspergillus* species and the resistance profile to azoles of *A. fumigatus* at national level and, in doing so, to contribute to informed decisions and policies on the control of aspergillosis and on the use of azole antifungals, both in medicine and in the environment.

## 2. Materials and Methods 

### 2.1. Aspergillus Identification and Diversity

During 2017 and 2018, *Aspergillus* isolates were obtained from clinical (respiratory, subcutaneous, or systemic) and environmental (soil, air, and surfaces from different sources, including hospitals, dwellings, and greenhouses) samples, analyzed at the Mycology National Reference Laboratory or received from collaborating institutions in the context of the *Aspergillus* surveillance program.

All isolates included in the surveillance program were assigned with a unique and sequential designation (VA1, VA2, etc.). All isolates were plated for growth as single colonies on malt extract agar with chloramphenicol (MEA). The morphological identification of the *Aspergillus* section was carried out based on macro and microscopic characteristics and using identification atlases [17,18].

Molecular identification of the species was carried out by partial sequencing the genes encoding calmodulin or beta-tubulin. Genomic DNA was prepared from each isolate using the High Pure PCR Template Preparation Kit (Roche Diagnostics GmbH, Mannheim, Germany) according to the manufacturer’s instructions.

Amplifications and sequencing of the partial beta-tubulin and calmodulin genes were performed as described previously [19]. The resultant nucleotide sequences were edited with Chromas Lite v 2.01 and aligned with CLUSTALX v 2.1 programs. Edited sequences were further compared with sequences deposited in the GenBank (Bethesda, MD, USA) and WI-KNAW Westerdijk Fungal Biodiversity Institute (Utrecht, The Netherlands) databases, in order to achieve the identification to species level, accepted when the obtained homology was ≥98%.

For both groups of clinical and environmental samples, species richness (total number of species), their abundance and diversity were evaluated through the Simpson diversity index (D = 1 − {[Σn(*n* − 1)]/[ΣN(*N* − 1)]}, where n is the total number of organisms of a particular species and N is the total number of organisms of all species; the index ranges from 0 to 1, the greater the value, the greater the diversity in the group), and the Shannon diversity index ($H^{\prime }=\Sigma pi\ln pi$, where *pi* is the proportion of individuals of species; the more unequal the abundance of species, the smaller the index; if abundance is primarily concentrated into one species, the index will be close to zero) [20].

### 2.2. Azole Susceptibility Profiling

For the study of azole-resistance patterns in *A. fumigatus* in Portugal, our entire 2012–2019 collection of isolates identified as belonging to *Fumigati* section was analyzed, which included both clinical and environmental isolates from different regions of the country.

Clinical isolates were obtained from biological specimens collected from different sites (mainly from the respiratory tract), from patients from 23 independent clinical institutions. Environmental isolates were collected from soil, air, and surfaces from previous referred sources (hospitals, dwellings, and greenhouses) but also from thermal spas, animal production farms, and a waste sorting plant. 

The pattern of susceptibility of the isolates to antifungal agents was carried out using an initial screening media for azole resistance. Plates of Sabouraud dextrose agar (SDA) (Oxoid, Hampshire, UK) supplemented with concentrations of 4 mg/mL of itraconazole (ICZ), 1 mg/mL (until 2017) [8] and 2 mg/mL (2018 to present) of voriconazole (VCZ) or 0.5 mg/mL of posaconazole (PCZ) were used [21,22,23]. Fresh conidia from a 7-day-old culture grown on MEA were suspended in saline solution at a turbidity equivalent to a 0.5 McFarland standard. Plates were inoculated by swabbing and incubated at 37 °C for 48 h. To control strain viability, an SDA plate without chloramphenicol was used. The reference strain *Aspergillus fumigatus* ATCC 204305 was used as negative control and a pan-azole resistant strain TR_34_/L98H (kindly provided by Professor Jacques Meis, Canisius-Wilhelmina Hospital, Nijmegen, The Netherlands) was used as positive control. *Aspergillus* growth was observed visually and classified as “Negative (−)” (no growth, susceptible), “Relevant growth (+)” (growth similar to positive control) and “Residual growth (±)” (growth of only one or few small colonies). Strains that were not able to grow in the azole-supplemented plates were considered as susceptible to the respective azoles, according to EUCAST guidelines [23]. In case of doubt or to confirm the screening results (for residual or relevant growths, respectively), the antifungal susceptibility profiles were determined by broth microdilution reference method for susceptibility testing of molds. The M38-A2 protocol from the Clinical and Laboratory Standards Institute was applied for determining the Minimal Inhibitory Concentrations (MIC) for ICZ, VCZ, PCZ. The final concentrations of drugs in the wells ranged from 0.0156 to 8 μg/mL for all. An internal control strain (*Aspergillus flavus* ATCC 204304) with known susceptibility was included as a positive control for MIC determination. SDA plates were inoculated with the final inoculum, incubated at 35 C, and examined after 48 h of incubation to check the inoculum concentration. Absence of visual growth defined the MIC. Isolates with high MICs were tested in triplicate to confirm the obtained results. Breakpoints for mold testing have not been established by the CLSI except for voriconazole and *A. fumigatus*. The CLSI epidemiological cut-off values (ECVs) used were 1 mg/L for ICZ, and 0.5 mg/L for PCZ and, for VCZ, 1 mg/L as breakpoint [24,25,26].

Working breakpoints were assigned according to Moin et al. [27] as follows: Itraconazole: susceptible (MIC ≤ 1 mg/L), intermediate (MIC = 2 mg/L), and resistant (MIC ≥ 4 mg/L); voriconazole: susceptible (MIC ≤ 1 mg/L), and resistant (MIC ≥ 2 mg/L); posaconazole: susceptible (MIC ≤ 0.5 mg/L), and resistant (MIC ≥ 1 mg/L). 

The AsperGenius^®^ Resistance multiplex real-time polymerase chain reaction (PCR) assay to detect TR_34_/L98H, and TR_46_/T289A/Y121F mutations and the AsperGenius^®^ G54/M220 RUO PCR assay to detect G54/M220 mutations (PathoNostics, Maastricht, The Netherlands) were used to test the isolates with a MIC for voriconazole, posaconazole, and/or itraconazole above the mentioned ECVs. These assays were performed on the Qiagen RotorGene Q instrument (Qiagen, Hilden, Germany) following the manufacturer’s instructions. To confirm the real-time PCR results and to check the existence of other mutations, point mutations, and tandem repeats, the *cyp51A* gene and its promoter were sequenced according to Prigitano et al. and Mellado et al. [28,29].

The programs GeneStudio ™ Professional Edition version 2.2.0.0 was used to edit the resultant nucleotide sequences and MEGA version 10.0.5 was used to align them. Edited sequences were compared with sequences deposited in the GenBank database (Bethesda, MD, USA).

## 3. Results

### 3.1. Molecular Epidemiology

From the 256 isolates collected during the study period (2017–2018), 156 were from clinical (human) sources and 99 from different environmental sources. 

One hundred and forty-four clinical isolates (92.3%) were from respiratory specimens, including bronchial/bronchoalveolar lavage (*N* = 109), bronchial secretions (*N* = 21), sputum (*N* = 12), and bronchial aspirate (*N* = 2). Other samples included chest drain pus (*N* = 1), tissue sample (*N* = 1), and clinical samples from unknown body source (*N* = 10). These clinical samples were collected from 129 patients (58 males, 66 females, 5 not known), with ages ranging from 17 to 88 years old at 14 hospital centers distributed throughout the country. The clinical information associated with the biological samples sent to the laboratory was scarce, but the majority of the patients presented bronchiectasis in their image exams (*N* = 32). Other clinical information available was: cavitary lung lesions/nodules/abscess/infiltrates (*N* = 11), respiratory infections/pneumonia (*N* = 9), HIV +/previous or active tuberculosis (*N* = 9); asthma (*N* = 2), invasive aspergillosis (*N* = 2), neoplasms (*N* = 3), cystic fibrosis (*N* = 1), chronic obstructive pulmonary disease (*N* = 1), diabetes (*N* = 1), and admission at the ICU, surgery, or infectiology units (*N* = 4). In 54 cases, no clinical information was provided to the laboratory.

*A. fumigatus* sensu stricto was the most frequent species in both clinical and environmental sources. Among the 156 clinical isolates, eight different *Aspergillus* sections were identified [*Fumigati* (*N* = 70), *Nigri* (*N* = 40), *Terrei* (*N* = 13)*, Flavi* (*N* = 11), *Circumdati* (*N* = 2)*, Clavati* (*N* = 1)*, Aspergilli* (*N* = 1), and *Nidulantes* (*N* = 13)], the latter now including previous section *Versicolores*) [30]. A total of 25 different species were identified (Table 1). 

Twenty clinical isolates were identified only as *Aspergillus* sp. by morphological methods; all were collected from respiratory samples (bronchial/ bronchoalveolar lavage and bronchial secretions) (Table 1). 

From those, 15 were identified by calmodulin and/or beta-tubulin sequencing, 11 of which were identified as cryptic species, some of them described as less susceptible to antifungals (as *A. lentulus*). In five isolates, no PCR product was obtained. Molecular identification to species level was obtained in 139 isolates. In total, cryptic species represented 36.7% (51/139) of the total clinical isolates. 

More than one *Aspergillus* species were isolated from the clinical samples of 19 patients (Table 2). Three patients (#6, #9, and #17) were infected/colonized by *A. fumigatus* sensu stricto and its cryptic species *A. lentulus.* This cryptic species was also isolated from patient #13, together with *A. niger* and *A. terreus*. Seven patients (#6, #7, #8, #10, #11, #13, and #19) were simultaneously colonized/infected with *A. terreus* together with other species. 

The 99 isolates were recovered from hospital environment (air, surfaces) (*N* = 17), soil (*N* = 1), air conditioning filters from different settings (*N* = 22), dwellings (air, surfaces) (*N* = 20), and air from agricultural environments (*N* = 19). Among these environmental isolates, eight different *Aspergillus* sections were identified [*Fumigati* (*N* = 39), *Nigri* (*N* = 21), *Usti* (*N* = 19), *Nidulantes* (*N* = 12), *Flavi* (*N* = 2), *Circundati* (*N* = 3), *Clavati* (*N* = 1), and *Flavipedes* (*N* = 2)]. Within these sections, 18 different species were identified (Table 1). Cryptic species represented 51.5% (50/99) of the total isolates. 

Species diversity was compared between clinical and environmental isolates. Simpson index showed greater species diversity in environmental samples compared with clinical samples (D = 0.82 versus D = 0.76, respectively). On the other hand, the abundance of a specific species is slightly greater in clinical samples when compared to environmental samples (Shannon index = −2.07 versus −2.12, respectively).

### 3.2. Characterization of the Resistance Pattern of Isolates from Fumigati Section

A total of 337 isolates belonging *Fumigati* section were collected in the period between 2012 and 2019 (Table 3). 

Among those, the frequency of cryptic species within this section was 5.3% (18/337). Twenty-four *Fumigati* isolates lost their viability and it was not possible to test them regarding their antifungal susceptibility. From the remaining, relevant and residual growth in azole-resistance screening media were obtained for 46 out of 296 sensu stricto isolates and for 7 out of 17 *Fumigati* cryptic species. Per published epidemiological cut-off values established using the CLSI M38A2 broth microdilution method, 4.4% of *A. fumigatus* sensu stricto isolates would be considered non-wild type to itraconazole (MIC >1 mg/L), 2.0% to voriconazole (MIC > 1 mg/L) and 2.0% to posaconazole (MIC > 0.5 mg/L) (Table 4).

Overall, all isolates considered as resistant or non-wildtype by microdilution grew in azole screening media (Table 4). It was not possible to run the MICs of two isolates (1 *A. fumigatus* sensu stricto and 1 *A. lentulus*), since they were not able to be recovered in culture.

Isolates that showed elevated MICs to azoles were screened by multiplex *Aspergillus* PCR kit for detection of specific mutations or sequenced the *cyp51A* gene and its promoter. 

The overall prevalence of resistance to azoles in *A. fumigatus* sensu stricto was 3.0% (four clinical—VA85, VA303, VA328, and VA346—and five environmental isolates—VA86, VA95, VA299CP, VA873CP, VA1209CP) (Table 4). Regarding clinical isolates, the amplification of the *cyp*51A gene of the VA85 isolate was inhibited, preventing any conclusions regarding the resistance mechanism involved (high MICs for PCZ and VCZ, intermediate for ICZ). Although displaying MICs higher than the proposed ECVs, no mutations were found in the *cyp*51A gene of VA328 isolate (high MICs for ICZ and PCZ, wild type for VCZ), whereas *cyp51A* sequencing of VA346 isolate (high MIC for PCZ, intermediate for ICZ, susceptible to VCZ) showed the presence of the following mutations: F46Y, M172V, N248T, D255E. In the VA303 isolate (pan-azole resistant), the TR_34_/L98H mutations were detected. For the environmental isolates considered as non-wild types, the TR_34_/L98H was also detected in the VA299CP, VA873CP, VA1209CP isolates, conferring pan-azole resistance. The multiplex *Aspergillus* PCR kit M220-G54 and the *cyp*51A gene sequencing allowed the detection of a G54R mutation in the VA86 isolate, conferring in vitro resistance to ICZ and PCZ. Although displaying MICs higher than the proposed ECVs for either ICZ, VCZ, and PCZ, no mutation was found in the *cyp51A* gene of the VA95 isolate.

The TR_34_/L98H was the most frequently detected mutation (in 1.4% of the isolates), found in three environmental and one clinical isolate. In all studied cases, data obtained by the real time *Aspergillus* multiplex PCR were corroborated by sequencing of the *cyp*51A gene and its promoter. 

Regarding only the *Fumigati* cryptic species, its overall resistance to azoles was 33.3% (all resistant isolates were from clinical sources—VA116, VA118, VA144, VA199, and VA254) (Table 4).

## 4. Discussion

In this study, we analyzed the *Aspergillus* distribution in clinical and environmental samples during a two-year period, from 2017 to 2018, and also the patterns of *A. fumigatus* azole-resistance using isolates from the surveillance program established by our Reference Laboratory, from 2012 to 2019.

Correct species identification within *Aspergillus* genus requires partial sequencing of the beta-tubulin or calmodulin genes, methods that are not available in most laboratories. This lack of identification to species level may have important consequences as cryptic species often display some level of intrinsic resistance to azoles and other antifungal drugs. Some of the clinical species identified during our study, such as *A. lentulus*, *A. udagawae*, *A. felis*, or *A. tubigensis* have been associated with refractory cases of IA [31,32,33].

As expected, *Fumigati* was the most frequently isolated section from our clinical samples. The same was observed in several epidemiological studies in other countries [5,34].

In a relevant percentage of cases, diagnosis of invasive aspergillosis is not done based on microbiological data and relies on suggestive imagology and/or positive fungal biomarkers such as galactomanan, β-1,3-glucan [35]. Hence, the actual contribution of *Aspergillus* spp. other than *A. fumigatus* (namely cryptic species) in clinical setting is unknown [36].

A high percentage (37%) of cryptic species was detected in our study in clinical isolates. To the best of our knowledge, this proportion of cryptic species of *Aspergillus* was the highest reported in literature, even doubling what was previously reported in a previous study from our group [19]. In another Portuguese study [16], this value was much lower (37.0% versus 7.5%). These differences may reflect the geographical source of the isolates and their epidemiological status. In both studies, the most frequent cryptic species was *A. welwitschiae*. In a study published by Pinto et al. [16], the 7.5% prevalence of cryptic species was distributed by the *A. niger* section (3.1%) and by the *A. fumigatus* section (2.2%). We found 84% of cryptic species among our clinical isolates belonging to *Nigri* section, and 5.2% belonging to the *Fumigati* section. In Spain, the percentage of clinical isolates identified as cryptic species belonging to *Fumigati* section was lower (2.2%) [37]. When observing fungal growth, several points should be observed to determine contamination, such as specimen type, amount and growth’s localization of the culture, pattern of growth, and number of cultures positive to the same organism [38].

Regarding mixed infections/colonizations, *A. terreus* was found frequently associated with other species, even with a pan-azole resistant *A. fumigatus* (patient #19). In a study published by Zoran et al. [39], posaconazole-resistance in *A. terreus* isolates is higher than 10%. Given the reduced susceptibility of *A. terreus* to amphotericin B [40], the management of those infections may be more difficult. Simultaneous colonizations/infections of *A. terreus* together with other species should be taken into consideration in the evaluation of therapeutic approaches. According to recent guidelines [41], species identification at the complex level should be carried out for clinically relevant isolates since some species are intrinsically resistant to either azoles or amphotericin B. Moreover, *Aspergillus* colonies from a single sample may harbor different resistance profiles and therefore it is advisable to perform multiple colony testing (up to five colonies) in order to increase sensitivity for azole-resistance detection [41].

Interestingly, several of the identified cryptic species, especially *A. lentulus*, were isolated together with *A. fumigatus* sensu stricto or with other species (Table 2). This has also been reported in other studies [42]. According to several authors [34,43,44,45], cryptic *Fumigati* species have been recognized as occasional causes of invasive aspergillosis in 3 to 6% of cases. Noteworthy, in our surveillance study, several of these cryptic species could not be identified by culture morphology, but only through molecular methods, and five isolates could not even be characterized at section level, having been referred as *Aspergillus* sp. (Table 1).

Infections due to *A. lentulus* or other *A. fumigatus*-related species are associated with a particularly high mortality rate, about 60% [36]. These species are often less susceptible to azoles [44,46], which is also reported in our study. Their actual prevalence may be underestimated because of their lack of recognition by conventional diagnostic approaches. Therefore, IDSA guideline on diagnosis and management of aspergillosis [47] recommends (strong recommendation and a high-quality evidence) that species identification by molecular methods should be performed in isolates with atypical growth or concerns for resistance.

Our results showed an even higher frequency of cryptic species in environmental isolates, as corroborated by Simpson index value. As already shown in several studies [48,49,50,51], the environment represents the major source of *Aspergillus* isolates causing infections. Exposure to *Aspergillus* may occur at home, in agricultural/animal production, at the workplace, and during periods of hospitalization, among others. The knowledge of the *Aspergillus* epidemiology of these environments may be important given that different fungal species may lead to different clinical manifestations and outcomes, depending on the host susceptibility, as well [52].

In 2017, the European Society of Clinical Microbiology and Infectious Diseases (ESCMID) recommend the susceptibility testing of *Aspergillus* on isolates causing invasive disease in patients from regions where resistance is found in surveillance programs [41]. However, this recommendation brings concern regarding the paucity of surveillance programs that perform mold susceptibility testing. Thus, this study aimed to present an estimate of the frequency of azoles resistant isolates of *A. fumigatus* in Portugal. According to our data, the estimated overall frequency of resistance in *A. fumigatus* sensu stricto was of 3.0%. Such a value differs from previous Portuguese studies. Amorim et al. [53] reported a <1% prevalence of itraconazole resistance in 159 isolates of *A. fumigatus* collected from cystic fibrosis patients receiving azole antifungal therapy. More recently, in a study on susceptibility of environmental *A. fumigatus* collected in the North of Portugal [54], 21.8% of isolates were resistant to itraconazole, 38.2% to posaconazole, and none of them were resistant to voriconazole. These differences may be explained by the date of collection of the isolates and by the geographical region from where the isolates were obtained.

According to a recent Spanish survey [37], 6.6% of patients carried azole-resistant *A. fumigatus* sensu lato. A similar percentage was obtained with our data, 4.8%.

The frequency of azole resistant *A. fumigatus* varies worldwide, ranging from 1.75% in India to 10%–20% in Europe, probably due to different usage of azole fungicides [27]. 

Azole-containing plates are easy to use in a routine mycology laboratory, offering the possibility of screening large collections of clinical strains at low cost [19]. When comparing the data obtained by azole screening with data obtained by the reference method, false negatives were rare (only three isolates with MIC > 0.5 mg/L that did not grow on PCZ-agar and one with MIC > 1 mg/L that did not grow on VCZ-agar). However, the multi-azole resistant isolates bearing the mutations TR_34_/L98H and G54R were detected in the screening media. According to Arendrup et al. [55], the sensitivity of azole screening media in detecting strains harboring the mutations G54E/R/V/W, G54R + N284K, M220I/K/T/V, M220I + V101F, TR_34_/L98H, and TR_46_/Y121F/T289A c*yp51A* mutations is 99%. 

On the other hand, false positives were frequent but in the majority of the isolates considered as having “residual” growth, MICs were below the ECVs values. Morio et al. [56] refer that the use of a concentration of 4 mg/L itraconazole, which is above the ECV for *A. fumigatus* might be a limitation, as some non-susceptible isolates could be missed. 

The Clinical Laboratory Standards Institute (CLSI) is developing susceptibility breakpoints for the triazoles and *A. fumigatus* to provide a tool to guide and optimize treatment. Recently, CLSI set breakpoints for voriconazole and *A. fumigatus*. These results identified voriconazole MIC values linked to treatment failure (≥2 μg/mL) and further identified susceptibility at concentrations ≤ 0.5 μg/mL. Regarding posaconazole, Espinel-Ingroff et al. [26] observed an overlap between MICs for non-mutant and mutant isolates was more evident with the ECVs of 0.5 μg/mL and, for that reason, we selected this value as ECV. Seven *A. fumigatus* sensu stricto isolates showed a MIC = 2 μg/mL to ICZ and three isolates showed a MIC = 2 μg/mL to VCZ. The latter value was considered as intermediate by CLSI. According to the most recent EUCAST guidelines [57], for some organism-agent combinations (as for *A. fumigatus* and itraconazole and voriconazole), results may be in an area designated the Area of Technical Uncertainty (ATU), where the interpretation is uncertain. In these cases, results should be reported as resistant with the comment that in some clinical situations (non-invasive infections) itraconazole can be used provided sufficient exposure is ensured. As an example, VA350 isolate showed MIC = 2 μg/mL to ICZ but no *cyp51A* mutation was found.

Among our *A. fumigatus* isolates collected from 2012 to 2019 and available for molecular testing, three harbored the TR_34_/L98H alterations in the *cyp51A* gene. These isolates were collected between 2018 and 2019, which may show an increase in the frequency of circulation of triazole-resistant *A. fumigatus.* The first clinical *A. fumigatus* isolates resistant to azoles detected in Portugal were described in 2018 [14]. According to our retrospective analysis, our first resistant isolate (mutation G54R) was collected in 2015, which reveals that azole resistant environmental isolates emerged in our country earlier than what was believed until now. Sharma et al. [58] also found resistant environmental isolates associated with this mutation. Isolate VA346 showed the presence of the following mutations: F46Y, M172V, N248T, D255E. These mutations (plus E427K) have been described to have different azole susceptibility profiles and to be azole susceptible or resistant, depending on the authors, but in all cases, they have higher azole MICs than *A. fumigatus* strains with wild-type *cyp51A* [59]. The same first four mutations were also found (together with E427K) in a pan-azole resistant Portuguese isolate analyzed by Monteiro et al. [54].

Mutation N248K was detected in two isolates that grew in azole screening media, but their MICs were lower than the proposed ECVs. As in other studies, N248K amino acid substitutions were previously reported in azole-susceptible *A. fumigatus*, suggesting that these alterations are SNPs that do not confer phenotypic resistance [60].

No mutations were detected in the *cyp51A* gene in the of the triazole-resistant VA95 isolate. As we have only screened for mutations on the *cyp51A*, it is possible that that resistance may be due to additional mechanisms of azole drug tolerance, such as mutations in *hapE* gene, decreased absorption of azole, or increased expression of efflux pumps [61]. 

Limitations of this study include the fact that the sequencing protocol used does not allow for the complete sequencing of the *cyp51A* gene (reaching only to the codon 365). Thus, mutations occurring at the end of this gene may not be detected. Furthermore, it was not possible to amplify calmodulin or beta-tubulin genes of several isolates, which resulted in an incomplete classification of our set of isolates. The isolates included in this study were not collected systematically from all regions of the country, which may lead to a bias in their geographical distribution. The majority of the isolates were obtained from samples that were not accompanied by enough clinical information, making the differentiation between colonization and infection difficult. Finally, when interpreting the resistance percentage reported here, we should also consider that, in clinical practice, a sizeable number of patients have culture-negative aspergillosis. As such, a positive selection bias toward resistance may occur, as sampling procedures are more likely to be undertaken for patients for whom antifungal therapy fails. Additionally, since the National Reference laboratory receives isolates voluntarily, a bias towards less common species (difficult to identify) and towards resistant isolates can occur, which can explain higher rates of cryptic species and may led to the overestimation of the resistance rate, in comparison to unbiased non-selected clinical isolates.

Considering the before mentioned limitations, it becomes clear that studies aiming at using a representative set of isolates and that may account for all these potential confounders are much needed, in order to have a more accurate estimation of the resistance profile in our country. 

In summary, this study highlights the importance of the implementation of a national surveillance network for *Aspergillus*. These findings are important contributions to raise the awareness to the importance of *Aspergillus* epidemiological distribution and to the surveillance of azole resistance in *A. fumigatus* in different Portuguese institutions, environments, and from diverse geographic locations. Understanding the prevalence of azole resistant isolates is important to guide clinical and public health decision-making.

## 5. Conclusions

The understanding shifts in the epidemiology of *Aspergillus* and of local resistance patterns is needed to manage therapeutic approaches. From all studied *Aspergillus*, a very high percentage of cryptic species was found. In our collection of *Fumigati* isolates, more than 5% of them were cryptic species and from those almost all showed resistance to azoles. From *A. fumigatus* sensu stricto, 3% showed resistance to azoles, being the TR_34_/L98H the most frequent mutation. 

## Figures and Tables

**Table 1:** Clinical and environmental *Aspergillus* species collected at the National Reference Laboratory in the scope of the *Aspergillus* surveillance program (2017–2018).

Section	Clinical Isolates			Environmental Isolates		
	Species	*N*	Total	Species (*N*)	*N*	Total
*Fumigati*	*A. fumigatus* sensu stricto	61	70	*A. fumigatus* sensu stricto	39	39
	*A. lentulus*	4				
	*A. felis/A. pseudofelis*	2				
	*A. udagawae*	2				
	Not identified to species level	1				
*Flavi*	*A. flavus* sensu stricto	8	11			2
	*A. sergii/transmontaneensis*	1				
	Not identified to species level	2				
				*A. nomius*	2	
*Nidulantes*	*A. nidulans* sensu stricto					12
	*A. delacroixii*					
	*A. minutus /insuetus*					
	*A. teneensis*			*A. teneensis*	1	
	*A. jensenii*					
	*A. fructus*	1		*A. fructus*	8	
	*A. tabacinus*	1		*A. tabacinus*	1	
	*A. creber/A. paulaauensis*	1				
	*A. versicolor sensu stricto/A. tabacinus*	1				
	Not identified to species level	1				
				*A. sidowii*	1	
				*A. protuberus*	1	
*Nigri*	*A. niger* sensu stricto	5	40	*A. niger* sensu stricto	5	21
	*A. welwitschiae*	22		*A. welwitschiae*	7	
	*A. tubigensis*	4		*A. tubigensis*	9	
	*A. brasiliensis*	1				
	Not identified to species level	8				
*Clavati*	*A. giganteus*	1	1			
				*A. clavatus* sensu stricto	1	1
*Terrei*	*A. terreus* sensu stricto	12	13			
	*A. alabamensis*	1				
*Circumdati*	*A. ochraceopetaliformis/A. flocculosus* (*N* = 1)	1	2			3
	*A. westerdijkiae* (*N* = 1)	1		*A. westerdijkiae*	1	
				*A. melleus*	2	
*Aspergilli*	*A. chevalierii* (*N* = 1)		1			
*Flavipedes*				*A. iizukae*	2	2
*Usti*				*A. ustus* sensu stricto	3	19
				*A. insuetus*	6	
				*A. minutus*	8	
				*A. germanicus*	2	
Not identified to section level	*Aspergillus* sp.		5			
**TOTAL**			**156**			**99**

**Table 2:** Patients infected/colonized by more than one *Aspergillus* species.

Patient #	Isolate	Product	Species
**1**	VA141	BAL	*A. fumigatus* sensu stricto
	VA142	BL	*A. welwitschiae*
**2**	VA151	BAL	*A. welwitschiae*
	VA152		*A. terreus* sensu stricto
**3**	VA153	BL	*A. fumigatus* sensu stricto
	VA154		*A. welwitschiae*
**4**	VA159	BAL	*A. welwitschiae*
	VA160	BL	*A. teneensis*
**5**	VA191	BAL	*A. fumigatus* sensu stricto
	VA192		*A. welwitschiae*
**6**	VA193	BAL	*A. terreus* sensu stricto
	VA194		*A. fumigatus* sensu stricto
	VA198	BL	*A. fumigatus* sensu stricto
	VA199		*A. lentulus*
**7**	VA228	BAL	*A. fumigatus* sensu stricto
	VA229		*A. terreus* sensu stricto
**8**	VA230	BL	*A. niger* (section)
	VA231		*A. terreus* sensu stricto
**9**	VA242	BAL	*A. lentulus*
	VA243		*A. fumigatus* sensu stricto
**10**	VA244	Respiratory sample	*A. fumigatus* sensu stricto
	VA245		*A. terreus* sensu stricto
**11**	VA251	Respiratory sample	*A. fumigatus* sensu stricto
	VA252		*A. terreus* sensu stricto
**12**	VA272	BL	*Aspergillus* sp.
	VA273		*A. fumigatus* sensu stricto
	VA274	BAL	*A. giganteus*
	VA275		*Aspergillus* sp.
**13**	VA276	BAL	*A. terreus* sensu stricto
	VA277	BAL	*A. lentulus*
	VA278		*A. niger* (section)
**14**	VA280	BL	*A. flavus* (section)
	VA289	BAL	*A. fumigatus* sensu stricto
**15**	VA281	BL	*A. flavus* sensu stricto
	VA285		*A. fumigatus* sensu stricto
**16**	VA296	Bronchial secretions	*A. fumigatus* sensu stricto
	VA297		*A. chevalierii*
**17**	VA298	Bronchial secretions	*A. fumigatus* sensu stricto
	VA299		*A. lentulus*
**18**	VA301	BAL	*A. niger* (section)
	VA304		*A. fumigatus* sensu stricto
**19**	VA302	BL	*A. terreus* sensu stricto
	VA303		*A. fumigatus* sensu stricto (TR_34_/L98H mutant)

Legend: BAL—Brochoalveolar lavage; BL—Bronchic Lavage.

**Table 3:** *Aspergillus* species from *Fumigati* section collected in the period 2012–2019.

Species	Number of Isolates	Source
*A. fumigatus* sensu stricto	319	Clinical and environmental
*A. lentulus*	8	Clinical (respiratory samples)
*A. felis/parafelis/pseudofelis*	5	Clinical (respiratory samples)
*A. hiratsukae*	1	Environmental (hospital environment)
*A. udagawae*	3	Clinical (respiratory samples)
*A. oerlinghauensis*	1	Clinical (ear exudate)

**Table 4:** Characterization of the isolates presenting growth on azole screening media.

											Azole Screening Media			Minimal Inhibitory Concentration (mg/L)			*cyp51A* Mutation
Isolate	Year Isolation	Geographic Location NUTS II	Location Code	Source	Biological Sample	Age	Gender	Clinical Information	*Aspergillus* Section	Molecular Identification	ICZ	VCZ	PCZ	ICZ	VCZ	PCZ	
VA7	2013	Lisbon	1	Clinical	Sputum	22	M	Cystic fibrosis	*Fumigati*	*A. fumigatus* sensu stricto	±	-	±	1	0.5	0.5	N.P.
VA8	2013	Lisbon	1	Clinical	Bronchial secretions	39	M	Cystic fibrosis	*Fumigati*	*A. fumigatus* sensu stricto	+	-	±	0.5	0.25	0.25	N.P.
VA9	2013	Lisbon	1	Clinical	Sputum	63	M	Cystic fibrosis	*Fumigati*	*A. fumigatus* sensu stricto	+	-	-	0.5	0.5	0.25	N.P.
VA10	2013	Lisbon	1	Clinical	N.R.	73	F	Cystic fibrosis	*Fumigati*	*A. fumigatus* sensu stricto	+	-	-	0.25	0.5	0.125	N.P.
VA16	2013	Lisbon	1	Clinical	Sputum	16	F	Cystic fibrosis	*Fumigati*	*A. fumigatus* sensu stricto	±	-	-	0.125	0.25	0.0625	N.P.
VA21	2013	Lisbon	1	Clinical	Bronchoalveolar lavage	48	M	Liver transplant	*Fumigati*	*A. fumigatus* sensu stricto	±	-	-	1	0.5	0.5	N.P.
VA35	2014	North	2	Clinical	Bronchial aspirate	67	F	N.R.	*Fumigati*	*A. fumigatus* sensu stricto	+	-	-	1	0.5	0.5	N.P.
VA37	2014	Lisbon	3	Environment	N.R.	N.A.	N.A.	N.A.	*Fumigati*	*A. fumigatus* sensu stricto	±	-	-	0.5	0.5	0.5	N.P.
VA44	2014	North	2	Clinical	Bronchial aspirate	79	M	Solid Tumor	*Fumigati*	*A. fumigatus* sensu stricto	±	-	±	0.5	0.5	0.5	N.P.
VA46	2014	North	2	Clinical	Bronchial aspirate	88	F	N.R.	*Fumigati*	*A. fumigatus* sensu stricto	±	-	±	0.5	0.25	0.5	N.P.
VA54	2015	Lisbon	4	Clinical	N.R.	N.R.	N.R.	N.R.	*Fumigati*	*A. fumigatus* sensu stricto	±	-	±	1	0.5	0.5	N.P.
VA55	2015	Lisbon	4	Clinical	Bronchial secretions	58	F	Chronic Liver disease	*Fumigati*	*A. fumigatus* sensu stricto	±	-	±	1	0.5	0.5	N.P.
VA63	2015	Lisbon	4	Clinical	Sputum	64	M	Sarcoidosis	*Fumigati*	*A. fumigatus* sensu stricto	±	-	±	2	0.5	0.5	N.P.
VA65	2015	Lisbon	4	Clinical	Bronchial secretions	21	M	Polytraumatized	*Fumigati*	*A. fumigatus* sensu stricto	±	-	-	0.5	0.25	0.5	N.P.
VA67	2015	Lisbon	4	Clinical	Sputum	55	M	Tuberculosis	*Fumigati*	*A. fumigatus* sensu stricto	±	-	±	0.5	0.25	0.5	N.P.
VA70	2015	Lisbon	4	Clinical	Bronchial secretions	78	M	Chronic obstructivepulmonary disease	*Fumigati*	*A. fumigatus* sensu stricto	±	-	-	1	0.5	0.5	N.P.
VA71	2015	Lisbon	4	Clinical	N.R.	N.R.	N.R.	N.R.	*Fumigati*	*A. fumigatus* sensu stricto	+	-	±	1	0.5	0.5	N.P.
VA73	2015	Lisbon	4	Clinical	Bronchoalveolar lavage	69	M	Diabetes	*Fumigati*	*A. fumigatus* sensu stricto	+	-	±	1	1	0.5	N.P.
VA74	2015	Lisbon	4	Clinical	Bronchial secretions	81	F	Sepsis	*Fumigati*	*A. fumigatus* sensu stricto	±	-	±	1	0.5	0.5	N.P.
VA76	2015	Lisbon	4	Clinical	Broncoalveolar lavage	41	F	Pulmonary emphysema	*Fumigati*	*A. fumigatus* sensu stricto	±	-	-	1	0.25	0.5	N.P.
VA77	2015	Lisbon	4	Clinical	Pulmonary tissue	57	M	HIV+	*Fumigati*	*A. fumigatus* sensu stricto	±	-	-	1	0.25	0.125	N.P.
VA78	2015	Lisbon	4	Clinical	Bronchial secretions	57	M	HIV+	*Fumigati*	*A. fumigatus* sensu stricto	-	-	±	1	0.5	0.5	N.P.
VA83	2015	Lisbon	4	Clinical	Peritoneal fluid	82	M	Liver cirrhosis	*Fumigati*	*A. fumigatus* sensu stricto	+	-	-	1	0.25	0.5	N.P.
VA85	2015	North	5	Clinical	Bronchial lavage	40	M	Tuberculosis and Aspergilloma	*Fumigati*	*A. fumigatus* sensu stricto	+	±	+	2	2	1	No amplification
VA86	2015	Centre	6	Environment	Swine air	N.A.	N.A.	N.A.	*Fumigati*	*A. fumigatus* sensu stricto	+	-	+	>8	0.5	2	G54R
VA95	2015	Lisbon	7	Environment	Air	N.A.	N.A.	N.A.	*Fumigati*	*A. fumigatus* sensu stricto	+	±	+	2	2	1	No mutation detected
VA137	2016	Lisbon	8	Clinical	Bronchial secretions	87	M	N.R.	*Fumigati*	*A. fumigatus* sensu stricto	±	-	±	1	0.25	0.125	N.P.
VA116	2016	North	2	Clinical	Bronchial secretions	70	F	N.R.	*Fumigati*	*A. lentulus*	+	±	+	2	8	1	N.P.
VA118	2016	North	3	Clinical	Sputum	80	M	Bronchiectasis	*Fumigati*	*A. lentulus*	+	+	+	2	>8	1	N.P.
HSMA67	2017	Lisbon	1	Environment	Hospital surface	N.A.	N.A.	N.A.	*Fumigati*	*A. fumigatus* sensu stricto	+	-	-	1	0.25	0.5	N.P.
VA146	2017	Leiria	9	Clinical	Bronchoalveolar lavage	45	M	HIV+	*Fumigati*	*A. fumigatus* sensu stricto	+	-	-	2	0.5	0.5	N.P.
VA149	2017	North	5	Clinical	Bronchoalveolar lavage	59	F	Bronchiectasis	*Fumigati*	*A. fumigatus* sensu stricto	+	-	-	1	0.5	0.5	N.P.
VA176	2017	North	6	Clinical	Bronchial lavage	67	M	Pulmonary infection	*Fumigati*	*A. fumigatus* sensu stricto	±	-	-	0.25	0.03	0.03	N.P.
VA144	2017	North	4	Clinical	Bronchial aspirate	57	M	N.R.	*Fumigati*	*A. felis*	+	+	+	4	>8	1	N.P.
VA182	2017	Lisbon	4	Clinical	N.R.	N.R.	F	HIV+, pulmonary aspergillosis, previous tuberculosis	*Fumigati*	*A. felis*	+	+	-	2	1	0.25	N.P.
VA199	2017	North	6	Clinical	Bronchial lavage	62	M	Cavitated pneumonia	*Aspergillus* sp.	*A. lentulus*	+	+	+	2	2	1	N.P.
VA244	2018	Lisbon	8	Clinical	Bronchial secretions	75	F	N.R.	*Fumigati*	*A. fumigatus* sensu stricto	±	±	-	1	1	0.5	N.P.
VA285	2018	North	6	Clinical	Bronchial lavage	83	F	Pulmonary abscess	*Fumigati*	*A. fumigatus* sensu stricto	±	-	-	1	0.25	0.5	N.P.
VA303	2018	North	6	Clinical	Bronchial lavage	65	M	Bronchiectasis	*Fumigati*	*A. fumigatus* sensu stricto	+	+	+	>8	8	2	TR_34_/L98H
VA242	2018	North	7	Clinical	Bronchoalveolar lavage	48	M	Bronchiectasis	*Aspergillus* sp.	*A. lentulus*	±	±	-	LV			N.P.
VA254	2018	Lisbon	7	Clinical	Ear exudate	14	F	N.R.	*Fumigati*	*A. oerlinghauensis*	+	+	-	4	4	2	N.P.
VA328	2019	North	6	Clinical	Bronchoalveolar lavage	84	M	N.R.	*Fumigati*	*A. fumigatus* sensu stricto	±	-	-	4	0.5	1	No mutation detected
VA346	2019	Centre	9	Clinical	Bronchial secretions	67	M	N.R.	*Fumigati*	*A. fumigatus* sensu stricto	±	-	-	2	0.5	1	F46Y, M172V, N248T, D255E
VA350	2019	North	6	Clinical	Bronchial lavage	56	M	Bronchiectasis	*Fumigati*	*A. fumigatus* sensu stricto	±	-	-	2	0.5	0.5	No mutation detected
VA299CP	2019	Alentejo	10	Environment	Cowshed air	N.A.	N.A.	N.A.	*Fumigati*	*A. fumigatus* sensu stricto	+	+	+	4	4	2	TR_34_/L98H
VA873CP	2019	Lisbon	11	Environment	FRPD fom waste sorting industry’ workers	N.A.	N.A.	N.A.	*Fumigati*	*A. fumigatus* sensu stricto	+	+	+	4	2	1	TR_34_/L98H
VA978CP	2019	Lisbon	11	Environment	FRPD fom waste sorting industry’ workers	N.A.	N.A.	N.A.	*Fumigati*	*A. fumigatus* sensu stricto	-	+	-	1	0.25	0.25	No mutation detected
VA1161CP	2019	Lisbon	12	Environment	Thermal SPA	N.A.	N.A.	N.A.	*Fumigati*	*A. fumigatus* sensu stricto	+	-	-	LV			N.P.
V1207CP	2019	Lisbon	11	Environment	FRPD fom waste sorting industry’ workers	N.A.	N.A.	N.A.	*Fumigati*	*A. fumigatus* sensu stricto	-	-	+	1	0.5	0.5	No mutation detected
VA1209CP	2019	Lisbon	11	Environment	FRPD fom waste sorting industry’ workers	N.A.	N.A.	N.A.	*Fumigati*	*A. fumigatus* sensu stricto	+	-	+	8	4	1	TR_34_/L98H
VA1215CP	2019	Lisbon	11	Environment	FRPD fom waste sorting industry’ workers	N.A.	N.A.	N.A.	*Fumigati*	*A. fumigatus* sensu stricto	-	+	+	1	0.25	0.125	N248K
VA1216CP	2019	Lisbon	11	Environment	FRPD fom waste sorting industry’ workers	N.A.	N.A.	N.A.	*Fumigati*	*A. fumigatus* sensu stricto	-	+	-	1	0.25	0.25	N248K
VA610CP	2019	North	13	Environment	Hospital air	N.A.	N.A.	N.A.	*Fumigati*	*A. fumigatus* sensu stricto	±	-	-	2	0.5	0.5	No mutation detected

N.A.: Not applicable; N.R.: Not referred; N.P.: Not performed; FRPD: Filtering respiratory protective devices; LV: Lost of strain viability.
